# Inflammatory findings on species extrapolations: humans are definitely no 70-kg mice

**DOI:** 10.1007/s00204-013-1038-0

**Published:** 2013-03-19

**Authors:** Marcel Leist, Thomas Hartung

**Affiliations:** 1 Doerenkamp-Zbinden Chair for In Vitro Toxicology and Biomedicine, University of Konstanz, Constance, Germany; 2 Doerenkamp-Zbinden Chair for Evidence-Based Medicine, Bloomberg School of Public Health, Johns-Hopkins University, Baltimore, MD USA

## Abstract

Modern toxicology has embraced in vitro methods, and major hopes are based on the Omics technologies and systems biology approaches they bring along (Hartung and McBride in ALTEX 28(2):83–93, 2011; Hartung et al. in ALTEX 29(2):119–28, 2012). A culture of stringent validation has been developed for such approaches (Leist et al. in ALTEX 27(4):309–317, 2010; ALTEX 29(4):373–88, 2012a; Toxicol Res 1:8–22, 2012b), while the quality and usefulness of animal experiments have been little scrutinized. A new study (Seok et al. 2013) now shows the low predictivity of animal responses in the field of inflammation. These findings corroborate earlier findings from comparisons in the fields of neurodegeneration, stroke and sepsis. The low predictivity of animal experiments in research areas allowing direct comparisons of mouse versus human data puts strong doubt on the usefulness of animal data as key technology to predict human safety.

Regulatory toxicology is involved with the prediction of human risk and with regulatory approaches to limit such ‘assumed/predicted’ risks to humans. This is a very particular form of science, in that it deals mostly not with facts (concerning human hazard), but with assumptions and predictions derived from models. For most compounds, the human hazard is (fortunately!) not known. The art of predictive toxicology lies in its construction of an intricate web of cross-relationships, to anchor the assumed human hazard to sets of real data. Animal experiments are to date the most important source of such data. A key question of the discipline is: how can we get information on the appropriateness of these data as anchor point for the toxicological ‘spider net’ of cross-references and extrapolations? ‘The proof of the pudding is the eating’, that is, human data are needed to control the validity of the prediction network.

The evaluation of whether animal experiments provide a solid starting point for the prediction of human hazard can follow two major lines. The first collects evidence from cases of human poisoning. At least for some compounds, this allows a direct comparison of effects on animals and on man. Prominent examples of case studies that suggest poor predictivity are the experience with thalidomide, or with the TG1412 drug candidate, which caused terrible effects in man that had not been predicted from the available animal data (Stebbings et al. 2007). Lack of correlation is also seen the other way around, that is, when rodent data predict cancer for compounds that are safe in man (Gold et al. 2005; Basketter et al. 2012). For some compound classes, there are also positive examples of animal data quantitatively predicting toxicity. However, in many areas of toxicology (for instance in the field of pesticides) such comparative data are hardly available. Moreover, this inductive approach (using individual case studies) does not allow conclusive general statements on the usefulness of animal experiments. Therefore, as a second line, deductive strategies to approach the question have been devised. Such approaches require answers to two types of question. For instance: (a) is there at least one field in which high-quality comparative data can be obtained? (b) can one show, or reasonably assume, that the predictivity of animals for man does not differ fundamentally in different fields of biomedical research? If answers to these questions can be obtained, a third step would be the combination of the answers for deduction of a generalized conclusion.

We will deal here with the second question only briefly. The answer from screening the scientific literature must be clearly ‘yes’. Tens of thousands of publications, all peer-reviewed, often in high-impact journals, are based on the assumption that animals are predictive of man in all the different research areas of animal use. Comparative claims that one area is particularly well or particularly badly predicted cannot be substantiated by the available scientific literature. Huge amounts of public money are spent on the assumption that animals are useful for all biomedical areas. No granting agency has ever declared a particular field of medical research to be pointless for animal-based research. Animals are applied uniformly as model in all areas of pharmacology, toxicology and general research in disease biology. This use is endorsed by committees of scientific experts, by ethical review boards, by the funding agencies and by political decision makers that channel the huge sums for funding of research and development into the different areas. The increasing use of animals for research in the last years has been accelerated by the widespread generation of transgenic mice. The increase in animal experimentation in most biomedical areas has overcompensated all successful efforts to substitute animals in some research fields (Hartung and Leist 2008; Blaauboer et al. 2012; Leist et al. 2012a; Hasiwa et al. 2011). The hard evidence for the belief in the usefulness of animal experimentation across fields (in terms of hundreds of millions of dollars and euros invested on basis of this assumption) is overwhelming.

This also relates to the field of toxicology, which cannot be separated from other biomedical research areas, as far as biological mechanisms and their correlation in man and animals are concerned (Leist et al. 2008a; Hartung 2009). Toxicology has profited a lot from findings and methods of other fields, and it is generally assumed that biochemical and physiological regulations, as well as their pathological counterparts discovered by different medical disciplines, do also apply to the field of safety sciences (Leist et al. 2008b; Rossini and Hartung 2012). We can thus safely assume that the predictivity of animal models is judged to be equally high in pharmacology and toxicology, and the following part will concentrate on where to find good comparative data of animals versus man.

An answer has been provided by a recent noteworthy study of Seok et al. (2013) from the ‘large scale collaborative research program on inflammation and the host response to injury’. They chose inflammation as a field of medical research, in which human data are available and in which the mouse models seem to have a very good mechanistic resemblance to the human disease situation. The biological response to injury was analyzed on a molecular level, by looking into the regulation of about 5,000 human genes relevant to inflammation and by comparing them to the murine counterpart responses. The result was surprising, almost shocking: the correlation was not only poor, it was virtually absent for the main study areas: burns, trauma, endotoxemia. When the study was expanded to other areas, such as sepsis and infection, poor correlations of human and mouse data were confirmed. Thus, responses in mice cannot predict human responses; at least in these fields. Based on the above considerations (question (b)), there is no reason to believe that the correlation would be better in any other field.

It might be argued, that this is only one study, and only one very particular and small field. In this context, it is important to look at the reasons, why these experiments were performed. The paper by Seok is not a stand-alone study, but it was triggered by worrying findings of 20 years of research, which suggested that non-predictive animal models might be the reason for the many clinical failures of new drugs in the field of sepsis. Sepsis is a systemic inflammatory response and still one of the leading causes of death on intensive stations worldwide. For this reason, enormous resources have been devoted to basic research into its mechanisms and to the discovery of drugs. Countless papers appeared in top-impact journals already in the 90 s, but translation of any animal finding into clinics failed. Opal and Cross (1999) summarized already then ‘It has become painfully evident that animal models provide misleading and overly optimistic estimates of the survival benefit of specific antisepsis drugs when compared to clinical efficacy in actual human sepsis’. This situation did not become better with more time for trials and optimization of animal studies (Buras et al. 2005). When the only treatment discovered by this approach, activated C-reactive protein had to be withdrawn from the market in 2011, more than 100 additional clinical trials had been performed, and it became evident that every single approach that had been successful in animals had failed (Rittirsch et al. 2007; Christaki et al. 2011). Nevertheless, animal-based studies in this field still continue to be financed. This somehow rings memories of how prince Hamlet’s behavior was described by Polonius: ‘Though this be madness, yet there is method in ‘t’.

Mice continue to be used as models, as their failure in the past has been claimed to be not due to a general inaptness of animal models, but rather to the poor quality, standardization and adaptation to clinical questions of such studies. It is in fact true that there is strong evidence for deficits in the quality and reporting of animal studies (Hartung 2008; Macleod and van der Worp 2010; Kilkenny et al. 2010; van der Worp et al. 2010; van der Worp and Macleod 2011). On this basis, one may ask whether the translational value (Hackam and Redelmeier 2006; Rice 2012) is high enough to justify further use.

Possibly, the poor correlation, and its connection with the quality of animal experimentation are particular features of research on inflammation and infection. To examine this, it is worth taking a look at an entirely different research field: ischemic stroke. It shares one important feature with inflammation research: the animal models are thought to be conceptually very close to the human situation. In human ischemic stroke, the blood circulation is occluded and exactly the same is modeled in animals. In endotoxemia, infection or burn injury, the stimuli in humans and mice are exactly the same. This is a favorable situation, compared to the fields of age-related neurodegeneration, cardiovascular disease, type II diabetes, asthma or cancer, which require generation of quite artificial animal models. Back to stroke: how well do the animal models work? They work similar as in inflammation: not at all. Apart from thrombolysis, every single neuroprotective treatment for cerebral ischemia that has worked successfully in animals (over 500 have been reported (van der Worp et al. 2010)) has failed in man. This negative statement is based on large numbers of trials, comprising dozens of large studies and hundreds of smaller ones (De Keyser et al. 1999; Gladstone et al. 2002; O’Collins et al. 2006; Savitz and Fisher 2007). Also, in this field, the failure has been attributed to the poor research standards, and quality criteria have been defined to amend this situation. Failure of drugs, despite adherence to such criteria, then triggered the design of new criteria (Dirnagl and Fisher 2012; Savitz and Fisher 2007). Also, in this respect, stroke research resembles inflammation research. The alternative conclusion, that animal studies are inherently not suited to predict the human situation, is considered more rarely (Musch et al. 2006; Matthews 2008).

Before a quick generalization of the conclusions, it is certainly prudent to take a closer look at adjacent research areas. A field related to inflammation and infection is the research that deals with ‘countermeasures to biological and chemical terrorism and warfare’. This example is highlighted here, as the department of defense of the US sponsored a National Academy of Science of the USA report on ‘animal models for assessing countermeasures to bioterrorism agents’, published in December 2011 (NRC 2011). The usefulness of animal models was evaluated by renowned scientists, and the conclusion of the report was that animal models would not be useful. Instead, a recommendation was issued that human cell-based 3D in vitro systems should be developed. This decision was taken so serious that altogether 200 Mio $ have been made available since for research in this field (Hartung and Zurlo 2012). Scientific areas related to the one of ischemic stroke are neurodegenerative diseases such as Alzheimer’s or Parkinson’s disease. This field has seen vast investments of the private and public sector. Dozens of neuroprotective drugs and principles have been discovered in animals, none of them ever worked in man.

Nevertheless, the belief that mouse research can result in information about human disease and its treatment is still held firmly by most scientific funding agencies. Findings on species differences tend to be neglected, and the skewing of the available data by a publication bias toward positive animal findings has only recently been unraveled (Sena et al. 2010). Just to name few examples, it has been clear before the Seok study that TLR4 signaling, a pivotal process in the inflammatory response, is different in man and mouse (Schmidt et al. 2010), and it is generally known that many inflammatory mediators take very different roles in different species. Even fundamental regulations ranging from neural control of airways (Schlepütz et al. 2012) to the biology of stem cells (Schnerch et al. 2010) are very different between species. All this evidence suggests that animals are not particularly good predictors of humans, in the areas where we have comparative data on different species. Is toxicology an exception? At least some comparative data are available from drugs that have been evaluated first in animals, then in man. The largest comparative study in this area (Olson et al. 2000) finds a poor (i.e., 43 %) predictivity of rodents for man. It is stated explicitly that this is not necessarily due to different metabolism, but possibly due to a different biology. Some examples for such molecular differences in toxicodynamics are well-known. For instance, man is about 1000-fold more sensitive to inhibition of the Na/K ATPase by the cardiac glycoside ouabain than mice (Kent et al. 1987), and the difference in sensitivity to bacterial endotoxin may even be in the million-fold range (Seok et al. 2013; Hasiwa et al. 2013). Thus, there are many individual examples suggesting that humans are not simply 70-kg mice, neither in pharmacology, nor in toxicology. The recent study of Seok et al. (2013) has corroborated this notion, based on a broad systematic approach. The statements of this paper have been endorsed by renowned scientists that have themselves relied on animal studies in the past. Their statement, on the failure of mice to predict for man in an important area of pharmacology, should be taken seriously—and also serve as food for thought in toxicology.
